# Cross-Fostering Increases Th1/Th2 Expression in a Prenatal Dexamethasone Exposure Rat Model

**DOI:** 10.1371/journal.pone.0115554

**Published:** 2014-12-19

**Authors:** Ho-Chang Kuo, Mindy Ming-Huey Guo, Shih-Feng Liu, Chih-Cheng Chen, Jiunn-Ming Sheen, Hong-Ren Yu, Mao-Meng Tiao, You-Lin Tain, Li-Tung Huang

**Affiliations:** 1 Department of Pediatrics, Kaohsiung Chang Gung Memorial Hospital, Kaohsiung, Taiwan; 2 Chang Gung University College of Medicine, Kaohsiung, Taiwan; 3 Department of Respiratory Therapy and Internal Medicine, Kaohsiung Chang Gung Memorial Hospital, and Chang Gung University College of Medicine, Kaohsiung, Taiwan; 4 Department of Traditional Chinese Medicine, Chang Gung University, Taoyuan, Taiwan; Mie University Graduate School of Medicine, Japan

## Abstract

**Background:**

Prenatal dexamethasone exposure has been reported to increase allergy potential in childhood possibly by interference with normal immunological development *in utero*. This study investigated the effects of prenatal dexamethasone on T helper cell immune responses in a rat model.

**Methods:**

Pregnant rats received either dexamethasone 0.1 mg/kg/day or normal saline from gestational day 14–21. Off-springs were cared for by their biological mother, or cross-fostered by the opposing group. Spleen and blood samples were collected at post-natal day 7 and 120 and tested for mRNA expression and plasma cytokine levels of Th1/Th2/Th17 immune response.

**Results:**

Both Th1 (T-bet) and Th2 (GATA-3) mRNA expression were shown to have a significant increase in the prenatal dexamethasone exposure group at day 120 (p<0.05). The plasma levels for Th1 (IFNγ and IL-2) and Th2 (IL-4, IL-5, IL-13) were found to have no significant differences between the two group (p>0.05). The mRNA expression of Th17 (RORγt) showed a significant decrease at post-natal day 120 as well as the plasma level of IL-17A at day 7 (11.21±1.67 vs. 6.23±1.06 pg/ml, p = 0.02). Cross-fostering by a dexamethasone exposed mother resulted in a significant increase in Th1/Th2 mRNA expression (p<0.05) and decrease of Th17.

**Conclusions:**

Prenatal dexamethasone exposure increased Th1, Th2 and decreased Th17 expression. Cross-fostering by a dexamethasone exposed mother results in more prominent increase of Th1 and Th2 expression.

## Introduction

The prevalence of allergic diseases including asthma, atopic dermatitis and allergic rhinitis has increased in recent decades primarily in developed countries [1], [2]. Recent increases of atopic diseases in children have been ascribed to both environmental and hereditary factors [3], [4]. In our previous study, maternal atopic history, rather than the paternal atopic history, was the major factor affecting infant eczema and cord blood immunoglobulin E (IgE) levels [5], [6]. These results suggest that maternal inheritance and the prenatal environment itself during pregnancy could be important determining factors in the development of atopic disease.

Prenatal dexamethasone is one of the most commonly used drugs during pregnancy and is routinely administered to women at risk of preterm delivery to advance fetal lung maturation. In vitro studies have shown that dexamethasone may inhibit Th1 immunity and promote Th2 immunity [7]. For example, naïve CD4+ T cells exposed to dexamethasone show increased levels of Th2-related IL-4 mRNA production[8] and were less sensitive to the effects of IL-12 [9], [10], a cytokine is needed for Th1 differentiation.

However there is a paucity of data that examines the effect of prenatal steroids on T-helper cell immunity. While a few retrospective population studies have found that antenatal steroid use is an independent risk factor for the development of asthma between 36 to 72 months of age [11], and later at 3 to 5 years of age [12]; this has not been validated in randomized control trials [13].

Th2 immunity appears to be the default phenotype in human neonates at birth [14], with Th1 immunity developing later in life. However, this normal shift in immunological development may be sensitive to exogenous toxins especially during certain critical periods of gestation [15]. For example, studies in rats have shown that lead exposure during the third trimester results in reduced Th1 related delayed-type hypersensitivity in adulthood [16].

It is possible that prenatal steroid exposure may similarly interfere with normal immunological development *in utero* To test the effect of prenatal steroids on Th1 and Th2 immunity, we designed the following rat model to gauge the effect of prenatal dexamethasone on Th1, Th2 and Th17 subset related mRNA expression and plasma cytokine levels in infancy (at post-natal day 7) and in adulthood (at post-natal day 120). Although both betamethasone and dexamethasone have been found to be equally effective in advancing fetal lung maturity [17], we chose dexamethasone in this study due to its wider clinical availability and cost-effectiveness [18].

## Material and Methods

### Ethics statement

Our animal protocol was reviewed and approved by the Institutional Animal Care and Use Committee (IACUC) of the Chang Gung Memorial Hospital (Approval Number 2012022902). Adult Sprague-Dawley rats (200–250 g, BioLASCO Taiwan Co., Ltd) were purchased from BioLASCO Taiwan Co.,Ltd. All animals were housed in an animal facility at 22°C, with a relative humidity of 55%, in a 12 h light/12 h dark cycle, with food and sterile tap water available ad libitum.

### Animal grouping

Virgin female rats were housed with male rats. According to our study protocol [19], female rats were separated from male rats 24 hours after mating, and then housed individually. Pregnant female rats were randomly assigned to either the dexamethasone exposure group or control group. Maternal rats in the control group were given intra-peritoneal normal saline injections once a day from gestational day 14 to 21. To conduct a prenatal dexamethasone exposure model, the experimental group was given intra-peritoneal injections of dexamethasone (0.1 mg/kg body weight) once a day from gestational age 14 to 20 [20], [21]. The dose of 0.1 mg/kg of dexamethasone was chosen according to previously established animal models of prenatal steroid exposure [22], [23]. To control for differences in postnatal environment, cross-fostering of offspring rats was performed. In all, offspring rats were split into four groups after birth: two groups were raised by their birth mother (control offspring raised by control group mothers, and dexamethasone group offspring raised by dexamethasone-exposed mothers), and two groups were cross-fostered after birth (control offspring raised by dexamethasone-exposed mothers and vice versa).

### Experimental procedures and specimen collection

Rats were sacrificed at post-natal day 7 and 120 respectively to assess acute and chronic effects of prenatal dexamethasone exposure during the infancy and adulthood. Both body weight and spleen weights were assessed. Blood samples were collected in heparin tubes; plasma samples were then extracted for cytokine level testing. Total RNA was extracted from spleen specimens using cultured cell total RNA Purification kit (Favorgen, cat. No. FABRK001-1). Both plasma and RNA samples were frozen at −80°C until use.

### Measurement of plasma cytokines levels associated with Th cell subset immunity using Luminex^200^ system

Measurement of the levels of cytokines within plasma collected at post-natal day 7 and 120 was performed using Luminex 200 system (Luminex, Austin, Tex.). Plasma concentrations of interleukin (IL) -2, interferon-γ (IFN-γ), IL-4, IL-5, IL-13, and IL-17A were assessed using the Milliplex Assay (Millipore) system. The study method was modified from previously reported methods [24], [25]. Antibody conjugated beads were incubated first with diluted standards or plasma from animal subjects for 2 hours and then with detector antibodies for 1 hour at room temperature. Fluorescent detection was performed after the sample had been incubated for 1.5 hours with biotin as reporter and incubated for 30 min with fluorescent dye-conjugated streptavidin-phycoerythrin. Cytokine levels were measured by using a flow cytometer and were analyzed with Flowmetrix software (Master Plex QT 1.2 system) [24].

### Real-time quantitative RT-PCR analysis of Th cell related mRNA expression

Reverse-transcription was performed using the High Performance Reverse Transcriptase System (EPICENTRE). The expression levels of T-bet, Gata3 and RORγt were detected by real-time RT-PCR using SYBR Green PCR Master Mix and ABI Prism 7500 Sequence Detection System (Applied Biosystems). The T-bet, Gata3 and RORγt expression levels were normalized using 18S rRNA as an internal control and were presented as absolute expression levels. The primers used for amplifying 18S rRNA were 5′-GTA ACC CGT TGA ACC CCA TT -3′ (forward), 5′- CCA TCC AAT CGG TAG TAG CG -3′ (reverse). The primers used for T-bet mRNA were 5′- TCC ACC CAG ACT CCC CAA CA -3′ (forward) and 5′- GGC TCA CCG TCA TTC ACC TCC A -3′ (reverse); for GATA-3 mRNA: 5′-CAC CCA GAC ACG CAC CAC CC -3′ (forward) and 5′-CGG CAT ACC TGG CTC CCG TG -3′ (reverse), and RORγt mRNA and 5′-CTG CAC TGT GTG AAG GGT GA -3′ and 5′-GAC AAG CCT TTT CTC CAT CG -3′ (reverse).

### Statistics

Comparison of continuous data (mean ± SE) was calculated by Student's *t* tests and/or Mann-Whitney *U* test. The median values of each parameter were used as cutoff values. A level of *p*<0.05 was accepted as statistically significant. All statistical tests were performed using SPSS 14.0 for Windows XP (SPSS, Inc, Chicago, USA).

## Results

### Th1 immune expression

**Figure 1:**
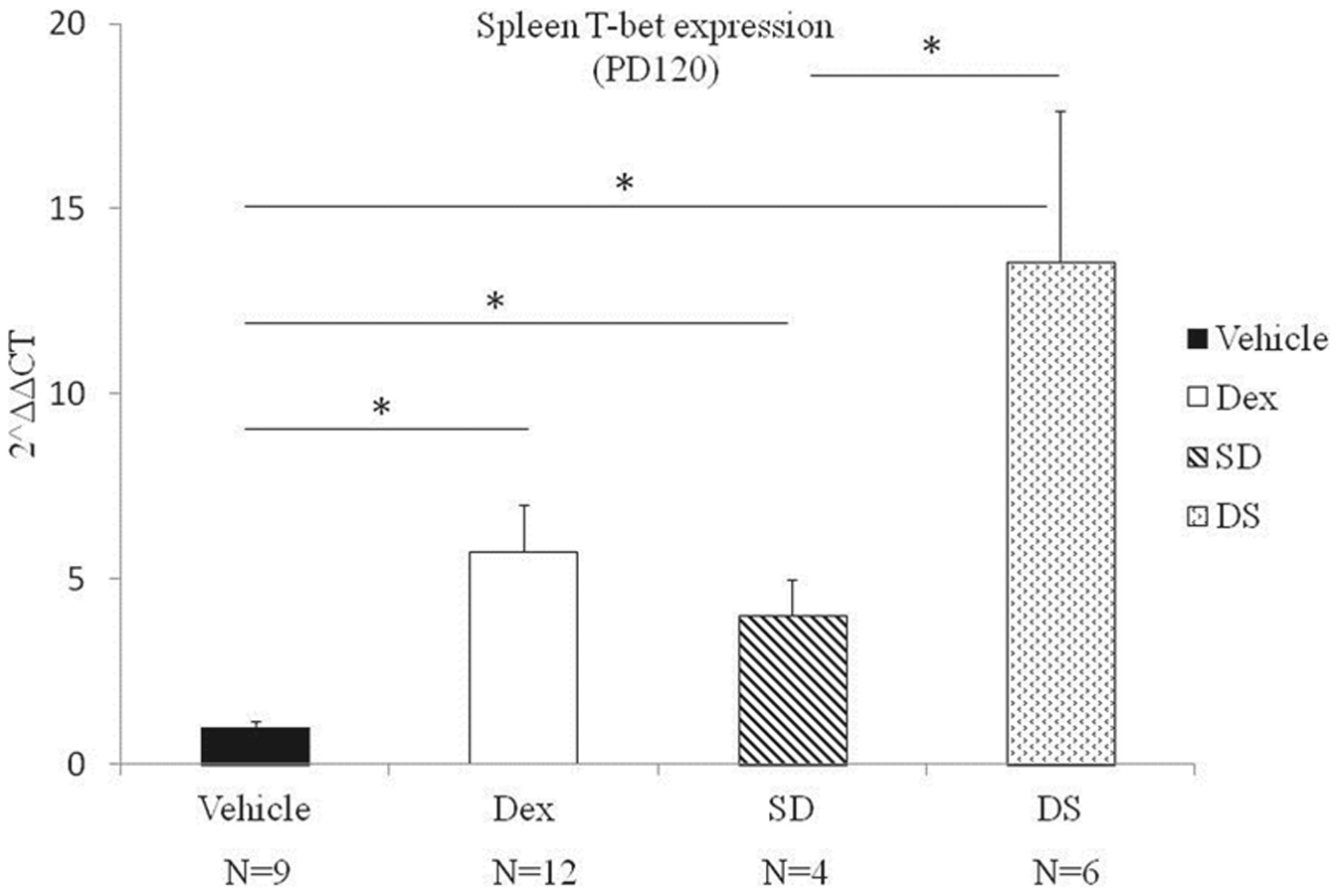
Th1 related mRNA expression levels. Animals were examined on post-natal day 120 (PD120). Data are expressed as mean ± SEM of 4–14 individuals. Vehicle: control group raised by control maternal rats, DEX: experimental group raised by experimental maternal rats, SD: Experimental group cross-fostered by control maternal rats, DS: Control group cross-fostered by experimental group maternal rats. An asterisk (*) Denotes a significant (p<0.05) between 2 groups by non-parametric tests.

Prenatal dexamethasone exposure had no significant effect on Th1 related mRNA T-bet expression at post-natal day 7 (p>0.05). The effects of prenatal dexamethasone exposure became more pronounced by post-natal day 120. In rats that were raised by mothers with no dexamethasone exposure, those who received prenatal dexamethasone had a higher level of expression of T-bet when compared with those who did not (Fig. 1, Dex vs. Vehicle groups, p<0.05).

There was no difference in plasma IL-2 and IFNγ levels at post-natal day 7 or post-natal day 120 (p>0.05). The effect of prenatal dexamethasone was found only on mRNA levels, but not cytokines levels.

### Th2 immune expression

**Figure 2:**
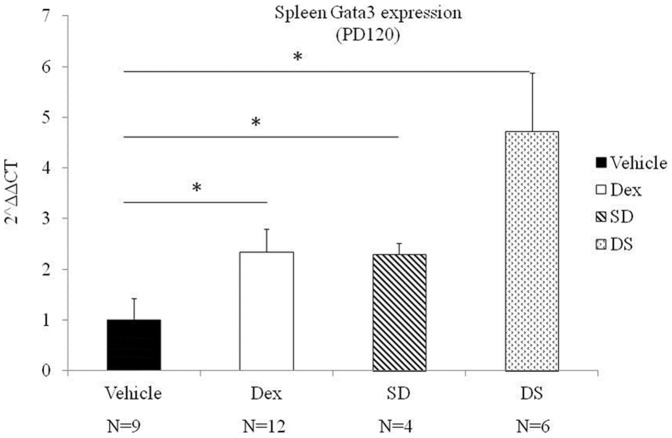
Th2 related Gata3 mRNA expression levels. Animals were examined on post-natal day (PD120). Data are mean ± SEM of 4–12 individuals. Vehicle: control group raised by control maternal rats; DEX: experimental group raised by experimental maternal rats, SD: Experimental group cross-fostered by control maternal rats, DS: Control group cross-fostered by experimental maternal rats. An asterisk (*) Denotes a significant (p<0.05) between 2 groups by non-parametric tests.

Prenatal dexamethasone exposure had no significant effect on Th2 related GATA3 mRNA expression levels at post-natal day 7 (Fig. 2a, *p*>0.05). By post-natal day 120, prenatal dexamethasone exposure was linked to higher expression of GATA3 mRNA (Fig. 2, Dex vs. Vehicle, *p*<0.05). Th2 related cytokines IL-4, IL-5 and IL-13 were all undetectable at post-natal day 7 and 120.

### Th17 immune expression

**Figure 3:**
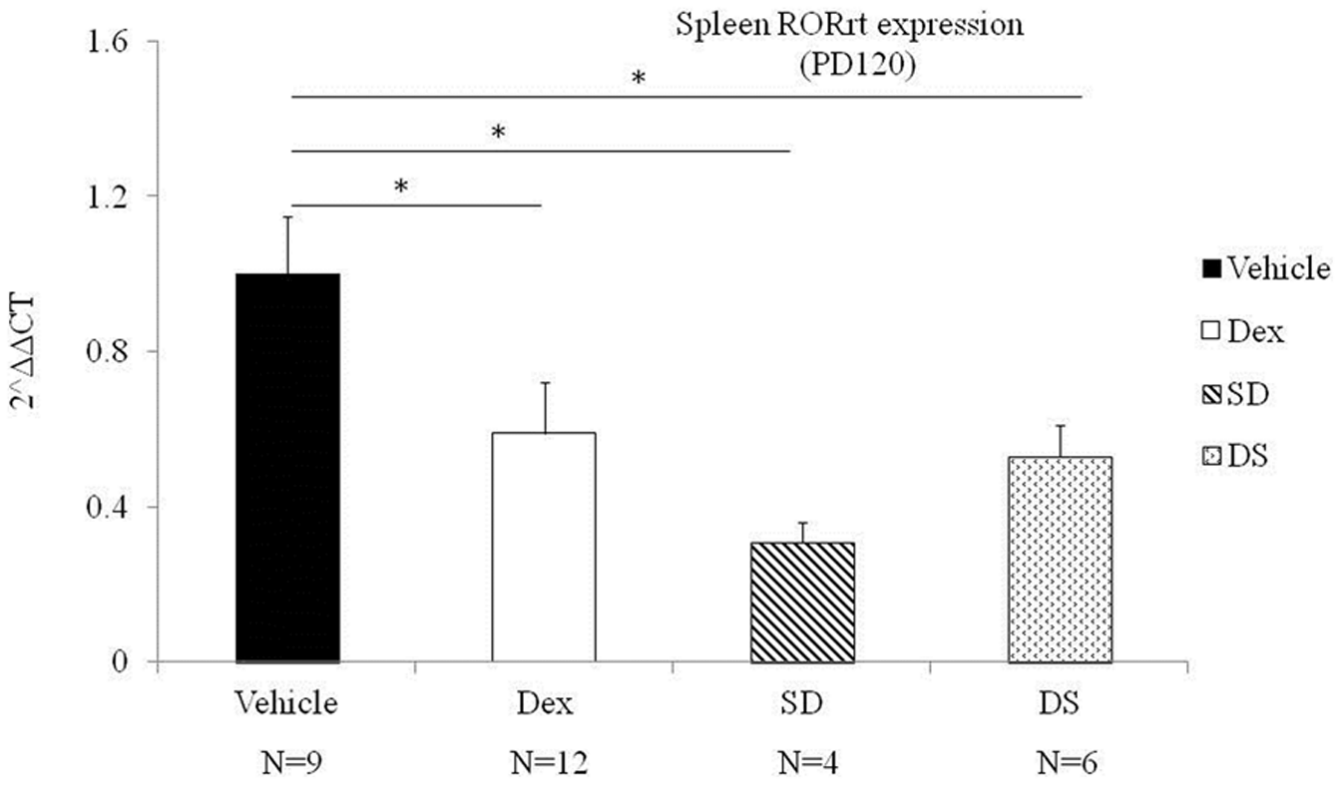
Th17 related mRNA and cytokine expression levels. Animals were examined on post-natal day (PD120). Data are mean ± SEM of 4–14 individuals. Vehicle: control group raised by control maternal rats, DEX: experimental group raised by experimental maternal rats, SD: Experimental group cross-fostered by control maternal rats, DS: Control group cross-fostered by experimental maternal rats. An asterisk (*) denotes a significant difference (p<0.05) between 2 groups by non-parametric tests.

There was no significant difference in RORγt mRNA expression at post-natal day 7 between the control and experimental groups (p>0.05). By post-natal day 120, rats that received prenatal dexamethasone had lower levels of RORγt mRNA expression when compared to those who did not (Fig. 3, Dex vs. Vehicle respectively, *p<*0.05). Prenatal dexamethasone exposure seems to have a corresponding effect in terms of Th17 related cytokine expression. Prenatal dexamethasone exposure was associated with decreased plasma levels of IL-17A at post-natal day 7 (11.21±1.67 vs. 6.23±1.06 pg/ml, P = 0.02, Mann-Whitney *U* test).

### The effect of cross-fostering on Th subset mRNA and cytokine expression

In rats with no prenatal dexamethasone exposure that were later cross-fostered by a dexamethasone exposed mother after birth, the effect of cross-fostering significantly increased the Th1 related T-bet mRNA, Th2 related GATA-3 mRNA, and decreased Th17 related RORγt mRNA expression at 120 days of life (Figs. 1, 2 and 3, p<0.05). Cross-fostering after birth did not affect IL-2, IFNγ, IL-4, IL-5, IL-13, IL-17A levels at 120 days after birth (p>0.05).

## Discussion

In our rat model, we found a consistent link between increased mRNA expression levels of T-bet and GATA3, the transcription factors related to Th1 and Th2 cells respectively, with a decrease in RORγt mRNA expression levels related to Th17 cells in the adult offspring (post-natal day 120) of rats with dexamethasone exposure during gestation. No changes in mRNA expression were noted early after birth at 7 days of age (post-natal day 7). In terms of plasma cytokine levels, prenatal dexamethasone exposure was related to a decrease in IL-17 plasma in infancy (post-natal day 7), but were undetectable by adulthood (120 days of life). Prenatal dexamethasone exposure did not affect the plasma levels of IL-2, IFNγ, IL-4, IL-5, and IL-13 levels at post-natal day 7 or 120. Overall, these results show that prenatal dexamethasone exposure is linked to increased Th1 and Th2 mRNA expression and suppression of Th17 related mRNA expression but no effect on cytokines levels.

These results suggest that exposure to dexamethasone *in utero* interferes with normal immune. In a study of a rat model by Dieter et al., prenatal dexamethasone produced a persistent functional loss of delayed type hypersensitivity, a Th1 immune response that was not evident in adults [26]. There has been a growing interest in the role of environmental insults *in utero* on immune development through epigenetic modifications. It is possible that prenatal dexamethasone may alter immunological expression through changes of DNA/histone methylation or acetylation [27], as opposed to direct interference of downstream transcription factors seen in previous studies [28], [29].

Although our study demonstrated an increase in both Th1 and Th2 related mRNA expression after prenatal dexamethasone exposure, there were no associated differences in cytokine concentrations. While there is no consensus as to whether mRNA expression or plasma concentrations are a more valid indicator of Th1 and Th2 responses, T-bet and GATA-3 are considered transcriptional factors specific to the development of Th1 and Th2 cells, respectively. Because Th1 and Th2 are related to cellular and humoral adaptive immunity, it is possible that the cytokine levels tested in our study represented baseline levels, but do not adequately reflect the immune response to external stimuli [8], [9]. In addition, post-transcriptional modifications or regulation at the mRNA level may account for why we found an increased expression of T-bet and GATA-3 mRNA, but found no difference in the related protein expression of IL-2, IFN-r, IL-4, IL-4 or IL-13 cytokines.

In the second portion of our study we found that cross-fostering by a dexamethasone exposed mother had an equal or possible more significant effect on Th subset mRNA expression. Rats with no prenatal dexamethasone exposure cross-fostered by a dexamethasone-exposed mother had increased levels of Th1 and Th2 related mRNA, and decreased levels of Th17 related mRNA at post-natal day 120; a change in that was identical to the one found in rats with prenatal dexamethasone exposure alone. In addition, cross-fostering by dexamethasone exposed mothers produced higher levels of Th1 and Th2 mRNA expression and lower levels of Th17 mRNA expression when compared to rats that were cross-fostered by mothers in the control group.

Previous studies have shown that prenatal dexamethasone alters pup-directed behavior in maternal rats [30]. In addition, cross-fostering in itself may present as a source of post-natal stress [31], resulting in endogenous production of stress related steroids in newborn rats. In our study we found that rats with no prenatal dexamethasone exposure cross-fostered by a dexamethasone-exposed mother (the DS group in Figs. 1 and 2) had the highest levels of Th1 and Th2 related mRNA expression. This may reflect the additive effects of both maternal exposure to dexamethasone and also the additional stress of cross-fostering.

Overall, our results seem to suggest that the post-natal environment had a larger effect on Th subset immunity when compared to the prenatal one. Postnatal factors involved in the development of Th subset immunity involve interplay of allergen exposure in the environment, diet, and exposure to infections and pollutants [32]. The effect of maternal prenatal dexamethasone exposure on Th mRNA expression in our study may be linked to differences in breast milk content, maternal behavior or allergens present in the maternal environment. Although prenatal glucocorticoids were linked to a decrease in milk volume in lactating mothers, [33] to date there has been no data published regarding the effect of prenatal steroids and changes in breast milk content or conferred immunity. Further studies are needed to examine the effect of prenatal dexamethasone on maternal behavior, breast milk content and the development of Th subset immunity.

### Conclusion

Our study demonstrates that prenatal dexamethasone exposure significantly increased Th1 and Th2 mRNA expression (T-bet and GATA-3), and decreased Th17 mRNA expression (RORγt) in adult offspring (postnatal day 120). A similar mRNA expression profile was also found in rats that were cross-fostered by a dexamethasone exposed mother. Further studies are needed to investigate both the net functional effect of prenatal dexamethasone exposure, and the underlying mechanisms involved in related mRNA expression.

## References

[1] Eder W, Ege MJ, von Mutius E (2006) The asthma epidemic. N Engl J Med 355:2226–2235. 17124020 10.1056/NEJMra054308

[2] Ronmark E, Bjerg A, Perzanowski M, Platts-Mills T, Lundback B (2009) Major increase in allergic sensitization in schoolchildren from 1996 to 2006 in northern Sweden. J Allergy Clin Immunol 124:357–363 363 e351–315.. 19577282 10.1016/j.jaci.2009.05.011 PMC2747664

[3] Bisgaard H, Simpson A, Palmer CN, Bonnelykke K, McLean I, et al (2008) Gene-environment interaction in the onset of eczema in infancy: filaggrin loss-of-function mutations enhanced by neonatal cat exposure. PLoS Med 5:e131. 18578563 10.1371/journal.pmed.0050131 PMC2504043

[4] Anderson WJ, Watson L (2001) Asthma and the hygiene hypothesis. N Engl J Med 344:1643–1644. 11374368 10.1056/NEJM200105243442116

[5] Liu CA, Wang CL, Chuang H, Ou CY, Hsu TY, et al (2003) Prenatal prediction of infant atopy by maternal but not paternal total IgE levels. J Allergy Clin Immunol 112:899–904. 14610477 10.1016/j.jaci.2003.08.030

[6] Yang KD, Ou CY, Hsu TY, Chang JC, Chuang H, et al (2007) Interaction of maternal atopy, CTLA-4 gene polymorphism and gender on antenatal immunoglobulin E production. Clin Exp Allergy 37:680–687. 17456215 10.1111/j.1365-2222.2007.02698.x

[7] Flammer JR, Rogatsky I (2011) Minireview: Glucocorticoids in autoimmunity: unexpected targets and mechanisms. Mol Endocrinol 25:1075–1086. 21511881 10.1210/me.2011-0068 PMC5417249

[8] Ramirez F, Fowell DJ, Puklavec M, Simmonds S, Mason D (1996) Glucocorticoids promote a TH2 cytokine response by CD4+ T cells in vitro. J Immunol 156:2406–2412. 8786298

[9] Wu CY, Wang K, McDyer JF, Seder RA (1998) Prostaglandin E2 and dexamethasone inhibit IL-12 receptor expression and IL-12 responsiveness. J Immunol 161:2723–2730. 9743329

[10] Fahey AJ, Robins RA, Kindle KB, Heery DM, Constantinescu CS (2006) Effects of glucocorticoids on STAT4 activation in human T cells are stimulus-dependent. J Leukoc Biol 80:133–144. 16670125 10.1189/jlb.0605296

[11] Pole JD, Mustard CA, To T, Beyene J, Allen AC (2009) Antenatal steroid therapy for fetal lung maturation: is there an association with childhood asthma? J Asthma 46:47–52. 19191137 10.1080/02770900802262795

[12] Pole JD, Mustard CA, To T, Beyene J, Allen AC (2010) Antenatal steroid therapy for fetal lung maturation and the subsequent risk of childhood asthma: a longitudinal analysis. J Pregnancy 2010:789748. 21490744 10.1155/2010/789748 PMC3065803

[13] Dalziel SR, Rea HH, Walker NK, Parag V, Mantell C, et al (2006) Long term effects of antenatal betamethasone on lung function: 30 year follow up of a randomised controlled trial. Thorax 61:678–683. 16601084 10.1136/thx.2005.051763 PMC2104681

[14] Prescott SL, Macaubas C, Holt BJ, Smallacombe TB, Loh R, et al (1998) Transplacental priming of the human immune system to environmental allergens: universal skewing of initial T cell responses toward the Th2 cytokine profile. J Immunol 160:4730–4737. 9590218

[15] Dietert RR, Lee JE, Bunn TL (2002) Developmental immunotoxicology: emerging issues. Hum Exp Toxicol 21:479–485. 12458904 10.1191/0960327102ht285oa

[16] Bunn TL, Parsons PJ, Kao E, Dietert RR (2001) Exposure to lead during critical windows of embryonic development: differential immunotoxic outcome based on stage of exposure and gender. Toxicol Sci 64:57–66. 11606801 10.1093/toxsci/64.1.57

[17] Brownfoot FC, Gagliardi DI, Bain E, Middleton P, Crowther CA (2013) Different corticosteroids and regimens for accelerating fetal lung maturation for women at risk of preterm birth. Cochrane Database Syst Rev 8:CD006764. 10.1002/14651858.CD006764.pub3 23990333

[18] Lawn JE, Kinney MV, Belizan JM, Mason EM, McDougall L, et al (2013) Born too soon: accelerating actions for prevention and care of 15 million newborns born too soon. Reprod Health 10 Suppl 1 S6. 24625252 10.1186/1742-4755-10-S1-S6 PMC3828574

[19] Tiao MM, Huang LT, Chen CJ, Sheen JM, Tain YL, et al (2014) Melatonin in the regulation of liver steatosis following prenatal glucocorticoid exposure. Biomed Res Int 2014:942172. 24822223 10.1155/2014/942172 PMC4005100

[20] Hauser J, Feldon J, Pryce CR (2009) Direct and dam-mediated effects of prenatal dexamethasone on emotionality, cognition and HPA axis in adult Wistar rats. Horm Behav 56:364–375. 19616002 10.1016/j.yhbeh.2009.07.003

[21] Tang JI, Kenyon CJ, Seckl JR, Nyirenda MJ (2011) Prenatal overexposure to glucocorticoids programs renal 11beta-hydroxysteroid dehydrogenase type 2 expression and salt-sensitive hypertension in the rat. J Hypertens 29:282–289. 21045727 10.1097/HJH.0b013e328340aa18

[22] Tain YL, Chen CC, Sheen JM, Yu HR, Tiao MM, et al (2014) Melatonin attenuates prenatal dexamethasone-induced blood pressure increase in a rat model. J Am Soc Hypertens 8:216–226. 24731552 10.1016/j.jash.2014.01.009

[23] Yu HR, Kuo HC, Chen CC, Sheen JM, Tiao MM, et al (2014) Prenatal dexamethasone exposure in rats results in long-term epigenetic histone modifications and tumor necrosis factor-alpha production decrease. Immunology 10.1111/imm.12346 PMC4253513 24962734

[24] Kuo HC, Wang CL, Liang CD, Yu HR, Huang CF, et al (2009) Association of lower eosinophil-related T helper 2 (Th2) cytokines with coronary artery lesions in Kawasaki disease. Pediatr Allergy Immunol 20:266–272. 19438983 10.1111/j.1399-3038.2008.00779.x

[25] Huang Y, Shan J, Zhang C, Zhang J, Feng L, et al (2010) Peripheral blood T regulatory cell counts may not predict transplant rejection. BMC Immunol 11:40. 20633262 10.1186/1471-2172-11-40 PMC2912834

[26] Dietert RR, Lee JE, Olsen J, Fitch K, Marsh JA (2003) Developmental immunotoxicity of dexamethasone: comparison of fetal versus adult exposures. Toxicology 194:163–176. 14636704 10.1016/j.tox.2003.07.001

[27] Martino DJ, Prescott SL (2010) Silent mysteries: epigenetic paradigms could hold the key to conquering the epidemic of allergy and immune disease. Allergy 65:7–15. 19796189 10.1111/j.1398-9995.2009.02186.x

[28] Liberman AC, Refojo D, Druker J, Toscano M, Rein T, et al (2007) The activated glucocorticoid receptor inhibits the transcription factor T-bet by direct protein-protein interaction. FASEB J 21:1177–1188. 17215482 10.1096/fj.06-7452com

[29] Liberman AC, Druker J, Refojo D, Holsboer F, Arzt E (2009) Glucocorticoids inhibit GATA-3 phosphorylation and activity in T cells. FASEB J 23:1558–1571. 19124555 10.1096/fj.08-121236

[30] Hauser J, Feldon J, Pryce CR (2006) Prenatal dexamethasone exposure, postnatal development, and adulthood prepulse inhibition and latent inhibition in Wistar rats. Behav Brain Res 175:51–61. 16956676 10.1016/j.bbr.2006.07.026

[31] Plyusnina IZ, Oskina IN, Tibeikina MA, Popova NK (2009) Cross-fostering effects on weight, exploratory activity, acoustic startle reflex and corticosterone stress response in Norway gray rats selected for elimination and for enhancement of aggressiveness towards human. Behav Genet 39:202–212. 19096923 10.1007/s10519-008-9248-6

[32] Peden DB (2000) Development of atopy and asthma: candidate environmental influences and important periods of exposure. Environ Health Perspect 108 Suppl 3 475–482. 10.1289/ehp.00108s3475 PMC1637811 10852847

[33] Henderson JJ, Hartmann PE, Newnham JP, Simmer K (2008) Effect of preterm birth and antenatal corticosteroid treatment on lactogenesis II in women. Pediatrics 121:e92–100. 18166549 10.1542/peds.2007-1107

